# Allelopathic Effects of Essential Oils on Seed Germination of Barley and Wheat

**DOI:** 10.3390/plants10122728

**Published:** 2021-12-11

**Authors:** Valtcho D. Zheljazkov, Ekaterina A. Jeliazkova, Tess Astatkie

**Affiliations:** 1 Crop and Soil Science Department, Oregon State University, Corvallis, OR 97331, USA; Ekaterina.Jeliazkova@oregonstate.edu; 2 Department of Engineering, Faculty of Agriculture, Dalhousie University, Truro, NS B2N 5E3, Canada; astatkie@dal.ca

**Keywords:** pre-harvest sprouting, *Triticum aestivum*, *Hordeum vulgare*, *Lavandula angustifolia*, *Hyssopus officinalis*, *Thymus vulgaris*, *Levisticum officinale*, *Chrysanthemum balsamita*, *Cuminum cyminum*, oil composition, seed germination

## Abstract

In this study, we evaluated the allelopathic effects of essential oils (EOs) from six different plant species, namely, lavender (*Lavandula angustifolia*), hyssop (*Hyssopus officinalis*), English thyme (*Thymus vulgaris*), lovage (*Levisticum officinale*), costmary (*Chrysanthemum balsamita*), and cumin (*Cuminum cyminum*), on seed germination and seedling growth of barley (*Hordeum vulgare*) and wheat (*Triticum aestivum*). The main constituents of the EOs of *L. angustifolia* were 47.0% linalool acetate and 28.4% linalool; *H. officinalis’* main constituents were 39.8% cis-pinocamphone, 9.8% trans-pinocamphone, 11.4% β-pinene, and 7.5% β-phellandrene; *T. vulgaris’* were 38.2% para-cymene, 25.6% thymol, and 13.6% γ-terpinene; *L. officinale’s* were 64.8% α-terpinyl acetate and 14.7% β-phellandrene; *C. balsamita*’s were 43.7% camphor, 32.4% trans-thujone, and 11.6% camphene; *C. cyminum’s* were 49.6% cumin aldehyde, 10.4% para-cymene, 11.6% α-terpinen-7-al, and 9.1% β-pinene. All six EOs exhibited an allelopathic effect and suppressed the seed germination and seedling development of wheat and barley; however, the concentrations that exhibited a suppressing effect were different among the plants. *C. cyminum* EO completely suppressed both barley and wheat germination at 10-, 30-, and 90-µL application rates, making it the most effective treatment among the tested EOs. *C. balsamita’s* and *H. officinalis’* EOs at 30 and 90 µL application rates completely suppressed barley and wheat radicles per seed, radicle length (mm), seedling height (mm), and germination (%). *L. angustifolia’s* EOs at 30- and 90-µL and *T. vulgaris’* EO at 90 µL application rates also completely suppressed barley and wheat radicles per seed, radicle length (mm), seedling height (mm), and germination (%). *C. balsamita’s*, *H. officinalis’*, *L. angustifolia’s*, and *T. vulgaris’* EOs at a 10 µL application rate reduced barley radicle length, seedling height, and % germination relative to the control. Wheat seed germination % was completely suppressed by the application of *L. angustifolia’s* and *T. vulgaris’* EOs at 30 and 90 µL, while *T. vulgaris’* EO at 10 µL rate reduced the germination relative to the control. Interestingly, *C. balsamita* and *H. officinalis* at 10 µL did not reduce wheat germination; however, they did reduce the number of radicles per seed, radicle length (mm), seedling height (mm), germination (%), and vigor index. Furthermore, *L. officinale’s* EO reduced the measured indices (radicles per seed, radicle length, seedling height, and vigor index) at the 10, 30, and 90 µL application rates relative to the non-treated control; however, none of the application rates of *L. officinale’s* EO had a suppression effect on wheat germination. This study demonstrated the allelopathic effects of the EOs of six different herbal plant species on seed germination of barley and winter wheat. The results can be utilized in the development of commercial products for controlling pre-harvest sprouting of wheat and barley. Further research is needed to verify the results under field conditions.

## 1. Introduction

Allelopathy in plants is either a beneficial or detrimental non-resource-based interaction between plants and is mostly related to the release of allelochemicals [1,2,3]. Allelopathy has been a subject of many studies [1] including in wheat, barley, and other Poaceae species [4,5].

Allelopathy can be utilized in modern agriculture to direct the interactions towards a desirable outcome for humans [6]. One such case is the suppression of pre-harvest sprouting in wheat and barley. Pre-harvest sprouting in these crops occurs as a consequence of the interaction between various factors [7,8,9,10]. Pre-harvest sprouting (also called vivipary, germination of seed on the mother plant) is germination of the grain in the ear head and caused by rainfall when the grain is ripe, but not yet harvested due to unfavorable weather. This is a major production issue that results in significant reduction of the yield and quality of wheat and barley that leads to loss of income. Surely, pre-harvest sprouting also depends on several other factors such as stage of grain maturity, dormancy and genetic resistance of the variety or cultivar, the amount and duration of rain, and the temperature at the time [9,10]. Plant essential oils (EO) have been known for their phytotoxicity that has been utilized for the development of more benign biopesticides [11] and for their allelopathic effects on seed germination.

The hypothesis of this study was that some EOs might be suitable for controlling pre-harvest sprouting in wheat and barley. If such EOs are identified, then these could be utilized in new-product development to control pre-harvest sprouting in wheat and barley fields. As of today, there are no chemicals that can be applied to large wheat or barley fields to prevent pre-harvest sprouting when rainy weather develops just before harvest. Therefore, the objective of this study was to evaluate the allelopathic effects of selected EOs from six different plant species on seed germination and seedling growth of wheat and barley. The long-term goal is to develop a product that can be feasibly applied to large areas with barley and wheat, when the conditions may be favorable for pre-harvest sprouting to occur.

The ideal product (1) would be environmentally friendly, not toxic for humans or the environment, (2) would suppress seed germination for a short period of time without killing the germ, (3) should be efficient in low concentrations to be applied with common pesticide applicator machinery including aircraft and helicopters, and (4) has short dissociation life span and is easily degradable.

## 2. Results

### 2.1. Chemical Profile of the Six Essential Oils (EOs)

The EO constituents of each of the EOs tested in this study are shown in Table 1. The main constituents of the EO were as follows: costmary: 43.7% camphor, 32.4% trans-thujone, 12% camphene, 2.6% bornyl acetate, 2.2% cis-thujone, 1.3% carvotanacetone, and 1.1% borneol; cumin: 49.6% cumin aldehyde, 11.6% α-terpinen-7-al, 10.4% para-cymene, 8.7% gamma-terpinen-7-al/thymol, 6% y-terpinene, and 9.1% β-pinene; hyssop: 39.8% cis-pinocamphone, 9.8% trans-pinocamphone, 11.4% β-pinene, 2.3% myrcene, 7.5% β-phellandrene, 2.5% β-caryophyllene, 3.1% germacrene D, 2.9% bicyclogermacrene, 2.1% myrtenyl methyl ether, 1.8% sabinene, 1.7% allo-aromadendrene, 1.7% elemol, 1.5% linalool, 1.2% β-bourbonene, and 1.0% myrtenol/methyl chavicol; lovage: 64.8% α-terpinyl acetate, 1.28% geranyl acetate, 1.7% α-pinene, 2.1% myrcene, 2.1% para-cymene, 14.7% β-phellandrene, 1.7% cryptone, 2.4% α-terpineol, and 1.1% cis-3-butylidene phthalide; lavender: 47.0% linalool acetate, 2.3% 3-octanone, 1.6% eucalyptol, 28.4% linalool, 2.5% lavandulyl acetate, and 2.5% caryophyllene oxide; and thyme: 38.1% para-cymene, 25.6% thymol, 13.6% γ-terpinene, 1.5% 4-terpineol, 2.2% β-caryophyllene, 2.5% caryophyllene, 1.3% α-thujene, 2.3% linalool, 2.1% myrcene, and 1.7% carvacrol (Table 1).

### 2.2. Effect of Six Essential Oils (EO) on Barley Seed Germination, Radicle, and Seedling Height

Barley median number of radicles per seed, radicle length (mm), seedling height (mm), germination (%), and vigor index obtained from the 11 treatments (other than *C. cyminum* 0 concentration) for barley are shown in Table 2. Representative photos of the germinating wheat and barley seeds in various treatments are shown in Figures S1 and S2.

The number of radicles per seed was significantly affected by the treatments. *C. balsamita’s* and *H. officinalis’* EO at 30 and 90 µL application rates completely suppressed radicles per seed, radicle length (mm), seedling height (mm), and germination (%) (Table 2). Both *C. balsamita’s* and *H. officinalis’* EO at a 10 µL application rate reduced radicle length, seedling height, and % germination relative to the control. *C. cyminum’s* EO completely suppressed germination at 10, 30, and 90 µL application rates, making it the most effective treatment. 

*L. angustifolia’s* EO at 30 and 90 µL and *T. vulgaris’* EO at 90 µL application rates also completely suppressed radicles per seed, radicle length (mm), seedling height (mm), and germination (%) (Table 3). *T. vulgaris’* EO at 30 µL significantly reduced % germination relative to the control, and completely suppressed radicle and seedling growth. At a 10 µL application rate, *L. angustifolia’s* and *T. vulgaris’* EO reduced the values of all response variables relative to the control. The % seed germination of barley was not suppressed by *L. officinale’s* EO 10 and 30 µL application rates; however, interestingly, the number of radicles per plant, radicle length, and seedling length were significantly reduced relative to the control, suggesting their effect on meristematic tissue and plant growth (Table 3). However, *L. officinale’s* EO at 90 µL suppressed the percent of barley germination and significantly reduced all other measured response variables relative to the non-treated control (Table 3).

### 2.3. Effect of the Six Essential Oils (EOs) on Wheat Radicles per Seed, Radicle Length (mm), Seedling Height (mm), Germination (%), and Vigor Index

As observed with barley, *C. balsamita’s* and *H. officinalis’* EOs at 30 and 90 µL application rates completely suppressed radicles per seed, radicle length (mm), seedling height (mm), and germination (%) of wheat (Table 4). Interestingly, *C. balsamita’s* and *H. officinalis’* at 10 µL did not reduce germination; however, they reduced the number of radicles per seed, radicle length (mm), seedling height (mm), germination (%), and vigor index. Similar to the effect on barley, *C. cyminum’s* EO at 10, 30, and 90 µL completely suppressed the measured responses in wheat (Table 4).

Furthermore, *L. angustifolia* and *T. vulgaris* at 30 and 90 µL application rates completely suppressed radicles per seed, radicle length (mm), seedling height (mm), and vigor index in wheat (Table 5). Additionally, at a 10 µL application rate, both EOs reduced the radicles per seed, radicle length (mm), seedling height (mm), and vigor index relative to the control. Furthermore, *L. officinale’s* EO reduced the measured indices (radicles per seed, radicle length (mm), seedling height (mm), and vigor index) at the 10, 30, and 90 µL application rates relative to the non-treated control (Table 5). Wheat seed germination % was completely suppressed by the application of *L. angustifolia’s* and *T. vulgaris’* EOs at 30 and 90 µL, while *T. vulgaris’* EO at 10 µL rate reduced the germination relative to the control. None of the application rates of *L. officinale* EO had a suppression effect on wheat germination (Table 6).

## 3. Discussion

This study is the first to evaluate the suppression effect of the vapors of the EOs of six herbal plant species (*C. cyminum*, *H. officinalis*, *C. balsamita*, *L. officinale*, *L. angustifolia*, and *T. vulgaris*) at four concentration levels (0, 10, 30, and 90 µL) on (1) Number of radicles per seed, (2) Radicle length (mm), (3) Seedling height (mm), (4) Germination (%), and (5) Vigor Index of wheat and barley. The results from this study demonstrated the allelopathic suppression activities of the vapors of these EOs on barley and wheat seed germination, radicle, and seedling growth.

A previous study demonstrated that the EOs of the plant species cumin, hyssop, costmary, lavender, and thyme may have potential for aphids’ control [12]. However, these EOs have not been tested for their allelopathic activity and, subsequently, their potential for being included in product developments for controlling pre-harvest sprouting in wheat and barley. Indeed, the EOs subject to this study were previously screened for their biological activities. The same EOs were shown to exhibit a repellency effect against *Rhopalosiphum padi* L. in a previous study and, hence, were considered promising for biological aphids’ control [12]. Furthermore, the EOs of these plant species have shown various other activities [13,14,15,16,17], including antimalarial activity [18], antifungal activity [19,20,21,22], and antibacterial activity [23,24].

There have been some reports on the allelopathic effect of extracts and EO of these plants on various other species.

### 3.1. Chrysanthemum balsamita (Tanacetum balsamita) and Its Essential Oil Allelopathy

There are not many reports on the use of *C. balsamita’s* allelopathic effect on seed germination. We were able to find one report indicating that distilled water or methanol extract from *Tanacetum balsamita* suppressed seed germination of large crabgrass (*Digitaria abscendens*) extremely well [25]. The application of *C. balsamita* distillation’s water (but not EO) on growing peppermint plants increased the EO content of peppermint [26].

Overall, the *C. balsamita* composition in this study was different from a literature report of the same species from Poland (Table 1) [27]. These variations may be due to differences in genotype as the *C. balsamita* in their study was wild-collected in Turkey and grown in a botanical garden in Poland [27].

### 3.2. Cuminum cyminum and Its Essential Oil Allelopathy

*Cuminum cyminum* EO has shown allelopathic effect on seed germination of the weed species of *Bromus tectorum*, *Centura ovina (most probably Centaurea ovina)* and *D. sophia*, which were completely inhibited by 2000, 1000 ppm, and 500 ppm, respectively [28]. In addition, *Cuminum cyminum* essential oils from 100 ppm to 1000 ppm decreased the germination percentage of *Bromus tectorum*, *Centaurea ovina*, and *D. sophia*. The authors concluded that *Cuminum cyminum* essential oils were allelopathic agents for weed control and should be a good agent for organic culture [28].

The overall composition of *C. cyminum* EO in this study was similar to that of a previous report (Table 1) [18], as in both cases the seed was obtained from the same supplier. In another study from Iran, the *C. cyminum* EO main compounds were reported to be 30.2% cumin aldehyde, 12.8% gamma-terpinene, and 6.4% beta-pinene [29]. These variations in the EO composition may be due to the genetic and environmental factors. 

### 3.3. Lavandula angustifolia and Its Essential Oil Allelopathy

*Lavandula angustifolia* EO has shown allelopathic effect on seed germination of seven Mediterranean weed species (*Amaranthus retroflexus* L., *Solanum nigrum* L., *Portulaca oleracea* L., *Chenopodium album* L., *Sinapis arvensis* L., *Lolium spp*., and *Vicia sativa* L.) [30]. However, cinnamon (*Cinnamomum zeylanicum* L.) EO was the most effective against the above weed species and completely inhibited the seed germination. *Lavandula angustifolia’s* EO was also shown to inhibit seedling emergence of the weed species *Amaranthus retroflexus* and *Portulaca oleracea* without much negative effect on tomato [31].

In another study, the *L. angustifolia* EO was tested against the germination and the radicle growth of the weed species *Raphanus sativus*, *Lactuca sativa*, and *Lepidium sativum* [32].

The EOs extracted from Lavandula and Salvia spp. showed good efficacy against *A. retroflexus* and *P. oleracea*, without severely affecting the tomato. Lavender EO has also shown phytotoxic (allelopathic) effects on *Vicia faba* root meristems [33]. Petrova et al. [34] evaluated the effect of water extract from the lavender flowers on wheat germination. However, this 24-h infusion in distilled water did not contain lavender EO and it is not clear what its composition was. Still, the application of 3.75% and 5% of this extract completely inhibited wheat seed germination [34]. Rossi et al. [35] evaluated the effect of EO of lavandin (*Lavandula x intermedia*), a species related to *L. angustifolia*, on insect in *Triticum durum* seed storage using the contact method by applying 1 mL of EO in methanol (50% v:v). The authors reported that the EO of lavandin at 50% completely inhibited wheat germination; however, its effect was low at an application rate of 1% [35].

The EO of *L. angustifolia* in this study contained 47% linalool acetate and 28% linalool (Table 1). This was similar to the composition of *L. angustifolia* grown in Mississippi, U.S., that had 26.5–40.5% linalyl acetate and 21–32% linalool depending on harvest and drying [15]. The latter authors also analyzed commercial lavender oils from Oregon, U.S.; France; and Bulgaria, and these samples showed 32–49% linalyl acetate and 25.1–43.4% linalool [15]. The *L. angustifolia’s* EO composition was also somewhat similar to the composition of the same species tested in Italy [32], which had 44.4% linalyl acetate and 23.1% linalool. In another study from England, *L. angustifolia’s* EO was found to contain 10–50% linalool and 26.5–40.5% linalool acetate [36].

### 3.4. Thymus vulgaris and Its Essential Oil Allelopathy

*Thymus vulgaris* EO has shown allelopathic effect and inhibition on seed germination of weeds and on wheat [37]. However, in the latter study, the EO was dissolved with distilled water to 0.3%, 0.6%, and 1% and the solutions were applied to the seed in Petri Dish, ensuring physical contact between the seeds and the solution. The *T. vulgaris* and *S. sclarea* oils alone and especially their blends showed promise as bioherbicides (to inhibit germination of weed species) against the seeds of the weed species *Amaranthus retroflexus*, *Chenopodium album*, and *Echinochloa crus-galli* control, but also reduced seed germination of wheat seed significantly [32]. However, the latter authors described that they dissolved the EO in distilled water prior to application; it is well known that EOs are not water soluble.

The study of the allelopathic effect of EOs on wheat and tomato seeds showed that the Salvia EO, applied at a level of 0.6%, resulted in a GI value of 88.4%, while a level of 1.0%, had the ability to inhibit the germination by 100% for tomato seeds and 80.5% for wheat seeds [37]. The EO of *Thymus vulgaris* was tested as a germination inhibitor for the seeds of three annual weeds (*Chenopodium album*, *Portulaca oleracea*, and *Echinochloa crus-galli*) and three crop species (*Raphanus sativus*, *Capsicum annuum*, and *Lactuca sativa*) [38]. The latter study’s experimental design utilized direct contact between the EO dissolved in water and Tween 20 and the seeds, while in this study, the seeds were exposed to the EO vapors. The *T. vulgaris* EO main constituents were 44% thymol, 21% p-cymene, and 11% gamma-terpinene [38]. Overall, the whole *T. vulgaris* EO showed better inhibition of seed germination of weeds and crop species than the pure compound thymol. The *T. vulgaris* EO reduced seed germination of *Raphanus sativus*, *Chenopodium album*, and *Echinochloa crus-galli* relative to the water control [38].

In another study, *T. vulgaris’* EO showed promise as a potential suppressor of seed germination and radicle growth of the weed species *Raphanus sativus*, *Lactuca sativa,* and *Lepidium sativum* [32]. The major EO constituents of *T. vulgaris* EO in this study were 38.2% para-cymene, 25.6% thymol, and 13.6% Gamma-Terpinene (Table 1), while the ones in the EO of the latter study were 56.2% o-Cymene, 24.4% carvacrol, and 8.7% thymol [32] (Table 1).

### 3.5. Levisticum officinale and Its Essential Oil Allelopathy

*Levisticum officinale* root infusion has shown inhibitory effect on germination of malting barley [39]; however, the latter report did not test the actual EO of *L. officinale*. The EO composition of *L. officinale* can be quite different depending on the plant part obtained from: whole herb, flowers, fruits ripe and non-ripe, or roots [40]. Therefore, it is important to report the plant part that was distilled for EO. In the latter study, β-Phellandrene ranged from 2.3% in roots to 48% in flowers [40]. Additionally, various studies demonstrated the effect of extraction on *L. officinale* EO composition. One such study of a wild population of *L. officinale* from Iran compared hydrodistillation vs. methanol extraction and reported significant differences in the concentrations of most EO constituents [41] (Table 1).

### 3.6. Hyssopus officinalis and Its Essential Oil Allelopathy

In the accessible literature, there was only one report on *H. officinalis’* EO on germination and the initial radicle growth of the weed species *Raphanus sativus*, *Lactuca sativa*, and *Lepidium sativum* [32]. The *H. officinalis* EO in the latter study [32] had 29.1% *iso*-Pinocamphone, 11.2% *trans*-Pinocamphone, and 18.2 *β*-Pinene, while the concentration of these compounds in the EO of our study was 39.8, 9.8%, and 11.4%, respectively (Table 1).

The EO composition of *H. officinalis* in this study was comparable to that of *H. officinalis* grown in Mississippi, U.S., that had 5.4 and 14.4% of β-pinene, 69.5 and 52.6% pinocamphene + isopinocamphene, and 1.1 and 2.2% myrcene in harvests 1 and 2, respectively [15]. The latter study also analyzed commercially available hyssop EO produced in Bulgaria, Canada, Europe, and Turkey and reported a β-pinene range of 7.8–17.4%, 11.9–25.5 pinocamphene + isopinocamphene, and 0.6–1.6 sabinene, demonstrating significant variation in hyssop EO composition depending on the production origin and probably due to variations in the E x G interactions [15]. In a study from Italy, the EO of *H. officinalis* grown at two different altitudes (100 m and 1000 m asl) had main EO constituents that were 34 and 18.5% pinocamphone, 3.2 and 29% isopinocamphone, 10.5 and 10.8% E-pinene, and 7.4 and 9.6% alpha-phellandrene, respectively [42], demonstrating a significant effect of the environment on the EO composition of this species.

## 4. Materials and Methods

### 4.1. Experimental Procedure

To study the effect of EO vapors on germination of barley and wheat seeds, an in vitro experiment was conducted in glass Petri dishes (100 mm × 15 mm). The EOs used in this study were obtained from cumin whole seed (*C. cyminum*), and from dried herbage of hyssop (*H. officinalis*), costmary (*C. balsamita*), lovage (*L. officinale*), English lavender (*L. angustifolia*), and English thyme (*T. vulgaris*) by Dr. Zheljazkov and colleagues via steam distillation.

### 4.2. Plant Material

The plant material selected for this study included a variety of species to represent widely available EO (lavender, hyssop, and thyme), less available and researched EOs (costmary, *C. balsamita*; and lovage, *L. officinale*), and also EO from different plant parts: seeds (cumin, *C. cyminum*), inflorescences (lavender and hyssop), and whole, aboveground plant parts (thyme, costmary, and lovage). Furthermore, these are grown as crops and the essential oils of these species are commercially available.

The plant material utilized to extract the EOs was obtained from various sources as follows: Certified bulk whole seed (*C. cyminum*) originated from India and was purchased from Starwest Botanicals (https://www.starwest-botanicals.com, accessed 3 December 2021/Sacramento, CA 95838, USA). The inflorescences of hyssop (*H. officinalis*) and English lavender (*L. angustifolia*), and the aboveground plant parts of costmary (*C. balsamita*), lovage (*L. officinale*), and thyme (*T. vulgaris*) were obtained from the experimental fields of the Research Institute for Roses and Medicinal Plants in Kazanluk, Bulgaria. These five crops were produced using certified seed lines maintained at the above Institute.

### 4.3. Extraction of the Essential Oils (EOs)

The EO of cumin seed was extracted via steam distillation for 240 min using 900 g of seed, in 2-L distillation units (Heartmagic, Rancho Santa Fe, CA, USA), as described previously [18]. The EOs from the other five plant species were extracted via steam distillation for 90 min using around 250 g of dried biomass material, as described previously for lavender and hyssop [15].

### 4.4. Gas Chromatography (GC) Analysis of the Essential Oils (EOs)

The EO samples from the six plant species (all oil samples in three replicates) were analyzed on a gas chromatograph (GC; Hewlett Packard Model 6890; Hewlett-Packard, Palo Alto, CA, USA) by Barry O’Brocki from Citrus and Allied, (https://citrusandallied.com, accessed 3 December 2021/4620 Mercedes Drive, Belcamp, MD 21017, USA), as described previously [43]. The carrier gas was helium at a flow rate of 40 cm·sec^−1^, 11.7 psi (60 °C), and a 2.5 mL·min^−1^ constant flow rate. The injection was split 60:1, 0.5 μL, and the injector temperature was 220 °C. The GC oven temperature program was as follows: 60 °C for 1 min and 10 °C.min^−1^ to 250 °C. The column was HP-INNOWAX (crosslinked polyethylene glycol; 30 m × 0.32 mm × 0.5 μm), and the flame ionization detector temperature was 275 °C.

Individual constituents of the EOs of cumin whole seed (*C. cyminum*), hyssop (*H. officinalis*), costmary (*C. balsamita*), lovage (*L. officinale*), English lavender (*L. angustifolia*), and thyme (*T. vulgaris*) are expressed as percentage of the total oil. The identification of individual constituent peaks was done with the use of internal standards by retention time and by mass spectroscopy.

### 4.5. Experimental Design

Four separate experiments were conducted: seeds of barley and wheat exposed to cumin whole seed, hyssop, or costmary EO vapors for 10 days and 11 days, respectively; seeds of barley and wheat exposed to lovage, English lavender, or English thyme EO vapors for 8 days and 10 days, respectively. The EOs were studied at the following concentrations: 0, 10, 30, and 90 μL. Seeds of barley or wheat were placed at the bottom part of the Petri dish on two layers of filter paper, and 5 mL of distilled water was added. To expose the seeds to EO vapors, the corresponding EO amount was added to a layer of filter paper placed on the inside of the lid. The lids were immediately closed, and each Petri dish was sealed with parafilm. The Petri dishes were placed into incufridges at 20 °C. To avoid producing an anaerobic environment inside the Petri dishes, the parafilm was removed after 24 h. The Petri dishes with the seeds were kept in the incufridges for the duration of the experiments. Seeds were monitored for germination approximately every-other day (Figure S1). At the end of each experiment, seedlings’ height and radicle length were measured. Vigor index was calculated by multiplying the percent of seed germination with the sum of seedlings’ height and radicle length and dividing by 100.

### 4.6. Statistical Analysis

The statistical analysis aimed at determining the significance of the main and interaction effects of EO (three levels: *C. cyminum*, *H. officinalis*, and *C. balsamita*) and EO concentration (four levels: 0, 10, 30, and 90 µL) for a duration of 10 days for barley and 11 days for wheat on (1) Number of radicles per seed, (2) Radicle length (mm), (3) Seedling height (mm), (4) Germination (%), and (5) Vigor Index. However, since there were several 0 response values, the normal distribution of the error terms’ assumption could not be met. Therefore, the Kruskal–Wallis nonparametric method that does not require normal distribution [44] was used by considering the 12 combinations of the factors as 12 treatments of a single factor with *n* = 4 replications. Since treatment effect was significant on all response variables, multiple medians comparison was conducted using the Mann–Whitney method for all pairs of treatments to generate letter groupings at the 5% level of significance.

However, the significance of the main and interaction effects of EO (three levels: *L. officinale*, *L. angustifolia*, and *T. vulgaris*) and EO concentration (four levels: 0, 10, 30, and 90 µL) for a duration of 8 days for barley and 10 days for wheat on (1) Number of radicles per seed, (2) Radicle length (mm), (3) Seedling height (mm), (4) Germination (%), and (5) Vigor Index was determined using a 3 × 4 factorial design with *n* = 4 replications. The validity of normal distribution and constant variance assumptions on the error terms were verified by examining the residuals, as described in Montgomery [45]. However, since most of the Germination (%) values for wheat were either 100% or 0%, no transformation could induce normality. Therefore, the nonparametric methods mentioned above were used. For all other response variables, the interaction between EO and EO concentration was highly significant. Since the experimental error was low (a lab experiment), Tukey’s multiple range test was conducted to compare the 12 combinations of EO and EO concentration at the 5% level of significance and generate letter groupings.

Both the nonparametric and parametric analyses were conducted using the NPAR1WAY and the GLM procedures of SAS 9.4, respectively [46]. 

## Figures and Tables

**Table 1:** Chemical composition of the essential oils of lavender (*Lavandula angustifolia*), hyssop (*Hyssopus officinalis*), English thyme (*Thymus vulgaris*), lovage (*Levisticum officinale*), costmary (*Chrysanthemum balsamita*), and cumin (*Cuminum cyminum*) on seed germination and seedling growth of barley (*Hordeum vulgare*) and wheat (*Triticum aestivum*).

Compound	RT [min]	Content [%] ± SD	Content from Literature [%]	Reference
------------------------------------ Costmary (*Chrysanthemum balsamita*) --------------------------------------------				
Cis-Salvene	5.17	0.16 ± 0.00		
Tricyclene	7.16	0.57 ± 0.00		
Camphene	8.05	11.63 ± 0.04	0.2; 0.02	[12,13]
Para-Cymene	10.35	0.25 ± 0.01		
Eucalyptol	10.62	0.49 ± 0.01	4.07	[13]
Camphenoline	12.49	0.08 ± 0.00		
Cis-Thujone	13.24	2.19 ± 0.02		
Trans-Thujone	13.84	32.43 ± 0.07		
Alpha-Campholenal	13.97	0.10 ± 0.00		
Camphor	14.96	43.72 ± 0.06	-	[13]
Cis-Chrysanthenol	15.37	0.12 ± 0.02		
Borneol	15.42	1.08 ± 0.01	0.3; 0.05	[12,13]
Myrtenol/Myrtenal	16.44	0.32 ± 0.01		
Carvotanacetone	18.18	1.27 ± 0.02	0.21	[13]
Cis-Chrysanthenyl Acetate	18.54	0.57 ± 0.01		
Bornyl Acetate	19.46	2.61 ± 0.00	-	[13]
Trans-Sabinyl Acetate	19.56	0.50 ± 0.01		
Isobornyl Isobutyrate	23.63	0.22 ± 0.00		
Isobornyl 2-Methyl Butyrate	26.37	0.20 ± 0.00		
Spathulenol	28.41	0.07 ± 0.00		
Phytone	34.23	0.15 ± 0.00		
Higher Alkane 1	45.85	0.10 ± 0.00		
Higher Alkane 2	49.5	0.06 ± 0.01		
---------------------------------------------- Whole Seed Cumin (*Cuminum cyminum*) ------------------------------------				
Cumene	7.19	0.04 ± 0.01		
Alpha-Thujene	7.24	0.20 ± 0.01		
Alpha-Pinene	7.49	0.44 ± 0.02	0.36; 0.6	[14,15]
Sabinene	8.71	0.36 ± 0.01		
Beta-Pinene	8.93	9.07 ± 0.21	7.95; 6.4	[14,15]
Myrcene	9.16	0.54 ± 0.01		
Para-Cymene	10.46	10.44 ± 0.23	14.1	[15]
Limonene	10.54	0.27 ± 0.01		
Beta-Phellandrene	10.58	0.08 ± 0.00		
Eucalyptol	10.64	0.14 ± 0.00		
Gamma-Terpinene	11.6	5.99 ± 0.16	5.59; 12.8	[14,15]
Cis-Sabinene Hydrate	11.83	0.05 ± 0.00		
Linalool/Trans-Sabinene	12.91	0.11 ± 0.01		
Nopinone	14.35	0.05 ± 0.00		
Trans-Pinocarveol	14.41	0.08 ± 0.00		
4-Terpineol	15.71	0.20 ± 0.00		
Alpha-Terpineol	16.16	0.10 ± 0.00		
Para-Menth-3-en-7-al	16.29	0.60 ± 0.01		
Myrtenol/Myrtenal	16.43	0.12 ± 0.01		
Cumin Aldehyde	18.36	49.61 ± 0.32	53.3; 30.2	[14,15]
Phellandral	19.17	0.15 ± 0.01		
Alpha-Terpinen-7-al	19.67	11.56 ± 0.15	13.3	[14]
Gamma-Terpinen-7-al/Thymol	19.85	8.71 ± 0.16	-	[15]
Para-Cymen-7-ol	19.91	0.20 ± 0.02		
Para-Mentha-1,4-dien-7-ol	20.85	0.11 ± 0.01		
10-Epi-Beta-Acoradiene	21.91	0.22 ± 0.02		
Carotol	25.54	0.13 ± 0.01		
------------------------------- Hyssop (*Hyssopus officinalis*) ------------------------------------				
Alpha-Thujene	7.25	0.29 ± 0.01	0.4	[16]
Alpha-Pinene	7.51	0.63 ± 0.01	1.0; 2.1	[16,17]
Camphene	7.99	0.13 ± 0.00	0.2	[16]
Sabinene	8.74	1.76 ± 0.02	1.4; 0.4	[16,17]
Beta-Pinene	8.95	11.43 ± 0.08	18.2; 5.4–14.4	[16,18]
1-Octen-3-ol	8.99	0.09 ± 0.01		
Myrcene	9.19	2.26 ± 0.02	1.8; 1.6–3.5; 1.1,2.2	[16,17,18]
Pseudolimonene/Alpha-Phellandrene	9.69	0.08 ± 0.00	T; 7.4–9.6	[16,17]
Alpha-Terpinene	10.1	0.10 ± 0.01	0.2; 0.7–1.9	[16,17]
Para-Cymene	10.38	0.26 ± 0.00	-	[16]
Beta-Phellandrene	10.63	7.51 ± 0.13	1.8	[16]
Eucalyptol	10.63	0.29 ± 0.07		
Cis-Ocimene	10.71	0.16 ± 0.01		
Trans-Ocimene	11.08	0.51 ± 0.00		
Gamma-Terpinene	11.52	0.18 ± 0.00	0.2	[16]
Cis-Sabinene Hydrate	11.83	0.17 ± 0.01	-	[16]
Terpinolene	12.56	0.10 ± 0.00	0.2	[16]
Linalool	12.88	1.49 ± 0.01	1; 0.2–7.9	[16,17]
Cis-Thujone	13.22	0.16 ± 0.02	0.1	[16]
Trans-Thujone	13.59	0.10 ± 0.00	-	[16]
Myrtenyl Methyl Ether	15.06	2.13 ± 0.01		
Trans-Pinocamphone	15.32	9.75 ± 0.03	11.2	[16]
Cis-Pinocamphone	16	39.8 ± 0.13		
Cryptone	16.15	0.19 ± 0.00		
Alpha-Terpineol	16.22	0.30 ± 0.01	0.1–0.5	[17]
Myrtenol/Methyl Chavicol	16.47	1.04 ± 0.01		
Linalyl Acetate	18.24	0.84 ± 0.01	0.3; 0.8–2.9	[16,17]
Lavandulyl Acetate	19.39	0.09 ± 0.01		
Methyl Myrtenate	19.8	0.09 ± 0.00		
Myrtenyl Acetate	20.71	0.14 ± 0.01	0.6	[16]
Neryl Acetate	21.81	0.06 ± 0.00		
Alpha-Copaene	22.44	0.15 ± 0.00	0.1	[16]
Beta-Bourbonene	22.81	1.21 ± 0.01	1.3	[16]
Beta-Elemene	22.9	0.20 ± 0.00	T	[16]
Methyl Eugenol	23.18	0.18 ± 0.01	0.7	[16]
Alpha-Gurjunene	23.59	0.46 ± 0.00	0.5	[16]
Beta-Caryophyllene	23.96	2.52 ± 0.01	1	[16]
Beta-Copaene	24.19	0.17 ± 0.00		
Aromadendrene	24.65	0.11 ± 0.01		
Isogermacrene D	24.75	0.16 ± 0.00		
Alpha-Humulene	24.96	0.50 ± 0.01	1.9–3.2	[17]
Allo-Aromadendrene	25.21	1.67 ± 0.01		
Germacrene D	25.8	3.11 ± 0.02	3.3–5.1	[17]
Bicyclogermacrene	26.25	2.92 ± 0.02	3.1; 1.4–1.6	[16,17]
Beta-Bisabolene	26.34	0.06 ± 0.00		
Gamma-Cadinene	26.64	0.15 ± 0.00		
Delta-Cadinene	26.85	0.13 ± 0.00		
Elemol	27.59	1.65 ± 0.02		
Spathulenol	28.43	0.41 ± 0.01	1.4–2.3	[17]
Caryophyllene Oxide	28.61	0.48 ± 0.01	-	[16]
Ledol	29.11	0.16 ± 0.01		
10-Epi-Gamma-Eudesmol	29.76	0.26 ± 0.01		
Tau-Cadinol	29.95	0.18 ± 0.00		
Beta-Eudesmol	30.27	0.17 ± 0.01		
Alpha-Eudesmol	30.32	0.27 ± 0.00		
---------------------------------------- Lavender (*Lavandula angustifolia*) -----------------------------------				
Cis-3-Hexenol	5.27	0.10 ± 0.01		
Tricyclene	7.49	0.07 ± 0.01		
Camphene	7.98	0.25 ± 0.01	0.1–0.2	[19]
Sabinene	8.7	0.06 ± 0.01	-	[16]
1-Octen-3-ol	8.74	0.17 ± 0.01		
3-Octanone	9.02	2.35 ± 0.03		
Myrcene	9.16	0.13 ± 0.02	0.3; 0.4–1.3	[16,19]
3-Octanol/Butyl Butyrate	9.26	0.20 ± 0.01		
Hexyl Acetate	9.82	0.49 ± 0.00		
Para-Cymene	10.35	0.16 ± 0.01		
Limonene	10.51	0.43 ± 0.02	0.3; 0.2–0.4	[16,19]
Eucalyptol	10.63	1.55 ± 0.02		
Cis-Linalool Oxide	12	0.88 ± 0.01	0.4	[16]
Trans-Linalool Oxide	12.54	0.83 ± 0.01		
Linalool	13.09	28.42 ± 0.19	23.1; 21–32; 10–50; 43	[16,18,19,20]
1-Octen-3-yl-ol	13.26	1.40 ± 0.01		
3-Octanol Acetate	13.64	0.28 ± 0.01		
Camphor	14.65	0.23 ± 0.02		
Isomethone	15.06	0.15 ± 0.01		
Borneol	15.37	0.70 ± 0.01	6.3; 1–4	[16,19]
Cryptone	16.01	0.44 ± 0.00		
Hexyl Butyrate	16.07	0.46 ± 0.05		
Alpha-Terpineol	16.15	0.51 ± 0.27	0.4	[16]
2,6-Dimethyl-3,7-Octadien-2,6-diol	16.17	0.38 ± 0.11		
Linalool Acetate	18.43	47.02 ± 0.25	44.4; 26.5–40.5; 12–54; 30	[16,18,19,20]
Lavandulyl Acetate	19.43	2.54 ± 0.01	0.1–14	[19]
Neryl Acetate	21.83	0.28 ± 0.00		
Geranyl Acetate	22.43	0.52 ± 0.00	-	[16]
Caryophyllene Oxide	28.62	2.48 ± 0.03	0.4; 3–8	[16,19]
-------------------------------------------- Lovage (*Levisticum officinale*) -----------------------------------------------				
Alpha-Thujene	7.24	0.14 ± 0.01	0.3	[21]
Alpha-Pinene	7.5	1.65 ± 0.03	0.6; 0.1	[21,22]
Camphene	7.98	0.70 ± 0.01	0.1; -	[21,22]
Beta-Pinene	8.85	0.60 ± 0.01	0.5; -	[21,22]
Myrcene	9.16	2.05 ± 0.05	2.3; 1.1	[21,22]
Pseudolimonene/Alpha-Phellandrene	9.68	0.35 ± 0.01	1.3	[21]
Alpha-Terpinene	10.1	0.18 ± 0.01	0.4	[21]
Para-Cymene	10.38	2.11 ± 0.02	0.8	[21]
Beta-Phellandrene	10.63	14.7 ± 0.23	12.9	[22]
Cis-Ocimene	10.7	0.24 ± 0.00		
Gamma-Terpinene	11.51	0.22 ± 0.00	7.8	[21]
Terpinolene/Para-Cymenene	12.56	0.19 ± 0.03	0.5	[21]
Linalool	12.84	0.13 ± 0.00	0.4	[21]
Cis-Par-Menth-2-en-1-ol	13.71	0.10 ± 0.00		
Pentyl Benzene	14.92	0.17 ± 0.02		
4-Terpineol	15.71	0.30 ± 0.01		
Naphthalene	15.96	0.12 ± 0.00	-	[21]
Cryptone	16.08	1.69 ± 0.02		
Alpha-Terpineol	16.2	2.40 ± 0.04	1.1; 0.8	[21,22]
Isobornyl Formate	17.88	0.13 ± 0.01		
Cumin Aldehyde	18.2	0.23 ± 0.00		
Bornyl Acetate	19.41	0.69 ± 0.01		
Par-Cymen-7-ol	19.66	0.21 ± 0.00		
Geranyl Formate	19.8	0.23 ± 0.00		
3-Oxo-Para-Meth-1-en-7-ol	21.25	0.36 ± 0.03		
Alpha-Terpinyl Acetate	21.77	64.8 ± 1.21	40.5; 52.4	[21,22]
Geranyl Acetate	22.45	1.28 ± 0.05		
Cis-3-Butylidene Phthalide	30.78	1.13 ± 0.02		
Lugustilide	32.26	0.95 ± 0.02		
---------------------------------------- Thymus (*Thymus vulgaris*) ----------------------------------------------------				
Alpha-Thujene	7.25	1.33 ± 0.01	T	[16]
Alpha-Pinene	7.5	0.99 ± 0.01	2.5; 1.2	[16,23]
Camphene	7.98	0.81 ± 0.01	1.2	[23]
Beta-Pinene	8.75	0.39 ± 0.01	-	[16]
1-Octen-3-ol	8.86	0.30 ± 0.01		
Myrcene	9.18	2.12 ± 0.01	0.1; 1.9	[16,23]
Alpha-Phellandrene	9.7	0.14 ± 0.00	T	[16]
Delta-3-Carene	9.91	0.13 ± 0.01	-	[16]
Alpha-Terpinene	10.12	0.95 ± 0.00	0.1	[16]
Para-Cymene	10.59	38.15 ± 0.10	56.2; 21.0	[16,23]
Limonene	10.64	0.56 ± 0.01	0.6; 0.5	[16,23]
Eucalyptol	10.66	0.19 ± 0.01		
Cis-Ocimene	10.72	0.25 ± 0.00		
Trans-Ocimeme	11.08	0.05 ± 0.00		
Gamma-Terpinene	11.66	13.63 ± 0.04	0.4; 10.5; 68.4	[16,23,24]
Terpinolene	12.58	0.17 ± 0.00	0.7	[16]
Linalool	12.91	2.29 ± 0.01	0.4; 2.5	[16,23]
Camphor	14.76	0.97 ± 0.01	T; 0.7	[16,23]
Borneol	15.37	0.80 ± 0.01	0.2; 2.0	[16,23]
4-Terpineol	15.76	1.49 ± 0.02		
Alpha-Terpineol	16.28	0.08 ± 0.00	0.3	[16]
Geraniol	18.48	0.10 ± 0.01	-;0.7	[16,23]
Thymol	19.8	25.61 ± 0.07	8.7; 44.1; 24.7	[16,23,24]
Para-Cymen-7-ol	19.84	0.58 ± 0.01		
Carvacrol	19.99	1.73 ± 0.01	24.4; 2.6	[16,23]
Thymol Acetate	21.58	0.07 ± 0.00		
Isobornyl Acetate	22.4	0.11 ± 0.00		
Beta-Bourbonene	22.79	0.05 ± 0.00	-	[16]
Beta-Caryophyllene	23.96	2.21 ± 0.01	0.1; 1.8	[16,23]
Alpha-Humulene	25.22	0.22 ± 0.01	T	[16]
Gamma-Cadinene	26.64	0.09 ± 0.00		
Delta-Cadinene	26.85	0.19 ± 0.00		
Caryophyllene	28.65	2.54 ± 0.01		

**Table 2:** Median number of radicles/seed, radicle length (mm), seedling height (mm), germination (%), and vigor index obtained from the 11 treatments (other than *Cuminum cyminum* 0 concentration) for barley. Within each column, medians sharing the same letter were not significantly different at the 5% level of significance.

EO of the Plant Species	Conc. (µL)	No of Radicles/Seed	Radicle Length (mm)	Seedling Height (mm)	Germination (%)	Vigor Index
*Chrysanthemum balsamita*	0	6.2 a	34.8 a	101.6 a	97.5 a	133.7 a
*Chrysanthemum balsamita*	10	3.8 ab	3.7 bc	3.6 bc	40.0 bc	3.4 bc
*Chrysanthemum balsamita*	30	0.0 b	0.0 c	0.0 c	0.0 c	0.0 c
*Chrysanthemum balsamita*	90	0.0 b	0.0 c	0.0 c	0.0 c	0.0 c
*Cuminum cyminum*	10	0.0 b	0.0 c	0.0 c	0.0 c	0.0 c
*Cuminum cyminum*	30	0.0 b	0.0 c	0.0 c	0.0 c	0.0 c
*Cuminum cyminum*	90	0.0 b	0.0 c	0.0 c	0.0 c	0.0 c
*Hyssopus officinalis*	0	6.1 a	31.2 a	106.5 a	100.0 a	136.7 a
*Hyssopus officinalis*	10	4.9 a	8.8 b	27.5 b	47.5 b	12.3 b
*Hyssopus officinalis*	30	0.0 b	0.0 c	0.0 c	0.0 c	0.0 c
*Hyssopus officinalis*	90	0.0 b	0.0 c	0.0 c	0.0 c	0.0 c

**Table 3:** Mean number of radicles/seed, radicle length (mm), seedling height (mm), germination (%), and vigor index obtained from the 12 combinations of EO and EO concentration (Conc.) for barley. Within each column, means sharing the same letter were not significantly different at the 5% level of significance.

EO of the Plant Species	Conc. (µL)	No of Radicles/Seed	Radicle Length (mm)	Seedling Height (mm)	Germination (%)	Vigor Index
*Lavandula angustifolia*	0	6.15 a	38.92 a	89.25 a	95.00 a	128.49 a
*Lavandula angustifolia*	10	1.40 d	1.58 cd	17.27 c	17.50 c	3.15 d
*Lavandula angustifolia*	30	0.00 e	0.00 f	0.00 d	0.00 d	0.00 d
*Lavandula angustifolia*	90	0.00 e	0.00 f	0.00 d	0.00 d	0.00 d
*Levisticum officinale*	0	5.45 ab	40.33 a	84.05 a	98.75 a	123.02 a
*Levisticum officinale*	10	6.35 a	13.77 b	52.00 b	88.75 a	59.81 b
*Levisticum officinale*	30	4.65 b	4.29 c	18.60 c	85.00 a	19.70 c
*Levisticum officinale*	90	2.60 c	1.33 d	6.85 d	50.00 b	4.50 d
*Thymus vulgaris*	0	6.35 a	35.75 a	86.90 a	96.25 a	117.97 a
*Thymus vulgaris*	10	0.40 de	0.11 e	1.00 d	6.25 cd	0.12 d
*Thymus vulgaris*	30	0.00 e	0.00 f	0.00 d	2.50 cd	0.00 d
*Thymus vulgaris*	90	0.00 e	0.00 f	0.00 d	0.00 d	0.00 d

**Table 4:** Median number of radicles/seed, radicle length (mm), seedling height (mm), germination (%), and vigor index obtained from the 12 treatments for wheat. Within each column, medians sharing the same letter were not significantly different at the 5% level of significance.

EO of the Plant Species	Conc. (µL)	No of Radicles/Seed	Radicle Length (mm)	Seedling Height (mm)	Germination (%)	Vigor Index
*Chrysanthemum balsamita*	0	5.3 a	89.6 a	110.5 a	100.0 a	197.9 a
*Chrysanthemum balsamita*	10	4.4 b	19.6 b	43.7 b	87.5 a	58.3 b
*Chrysanthemum balsamita*	30	0.0 c	0.0 c	0.0 c	0.0 b	0.0 c
*Chrysanthemum balsamita*	90	0.0 c	0.0 c	0.0 c	0.0 b	0.0 c
*Cuminum cyminum*	0	5.5 a	72.5 a	112.5 a	100.0 a	181.1 a
*Cuminum cyminum*	10	0.0 c	0.0 c	0.0 c	0.0 b	0.0 c
*Cuminum cyminum*	30	0.0 c	0.0 c	0.0 c	0.0 b	0.0 c
*Cuminum cyminum*	90	0.0 c	0.0 c	0.0 c	0.0 b	0.0 c
*Hyssopus officinalis*	0	5.7 a	71.8 a	114.3 a	100.0 a	187.7 a
*Hyssopus officinalis*	10	4.6 b	21.1 b	45.6 b	90.0 a	60.6 b
*Hyssopus officinalis*	30	0.0 c	0.0 c	0.0 c	0.0 b	0.0 c
*Hyssopus officinalis*	90	0.0 c	0.0 c	0.0 c	0.0 b	0.0 c

**Table 5:** Mean number of radicles/seed, radicle length (mm), seedling height (mm), and vigor index obtained from the 12 combinations of EO and EO concentration (Conc.) for wheat. Within each column, means sharing the same letter were not significantly different at the 5% level of significance.

EO of the Plant Species	Conc. (µL)	No of Radicles/Seed	Radicle Length (mm)	Seedling Height (mm)	Vigor Index
*Lavandula angustifolia*	0	5.2 ab	64.0 a	84.3 a	148.3 a
*Lavandula angustifolia*	10	4.9 ab	29.2 b	36.9 c	64.5 b
*Lavandula angustifolia*	30	0.0 e	0.0 c	0.0 e	0.0 d
*Lavandula angustifolia*	90	0.0 e	0.0 c	0.0 e	0.0 d
*Levisticum officinale*	0	5.4 a	61.2 a	80.4 ab	141.6 a
*Levisticum officinale*	10	5.0 ab	38.3 b	43.0 c	81.3 b
*Levisticum officinale*	30	4.6 bc	5.1 c	14.1 d	19.1 c
*Levisticum officinale*	90	2.7 d	2.1 c	5.9 de	8.0 cd
*Thymus vulgaris*	0	5.4 a	61.2 a	72.9 b	134.1 a
*Thymus vulgaris*	10	3.9 c	3.6 c	9.0 de	4.6 cd
*Thymus vulgaris*	30	0.0 e	0.0 c	0.0 e	0.0 d
*Thymus vulgaris*	90	0.0 e	0.0 c	0.0 e	0.0 d

**Table 6:** Median germination (%) obtained from the 12 treatments for wheat. Medians sharing the same letter were not significantly different at the 5% level of significance.

EO Species	EO Conc. (µL)	Germination (%)
*Lavandula angustifolia*	0	100.0 a
*Lavandula angustifolia*	10	100.0 a
*Lavandula angustifolia*	30	0.0 c
*Lavandula angustifolia*	90	0.0 c
*Levisticum officinale*	0	100.0 a
*Levisticum officinale*	10	100.0 a
*Levisticum officinale*	30	100.0 a
*Levisticum officinale*	90	100.0 a
*Thymus vulgaris*	0	100.0 a
*Thymus vulgaris*	10	27.5 b
*Thymus vulgaris*	30	0.0 c
*Thymus vulgaris*	90	0.0 c

## Data Availability

Data is contained within the article and supplementary material.

## Supplementary Materials

The following are available online at https://www.mdpi.com/article/10.3390/plants10122728/s1. Figure S1: Representative images of germinating wheat in various treatments using the essential oils. Figure S2: Representative images of germinating barley in various treatments.
