# Supplementary material for: Allelopathic Effects of Essential Oils on Seed Germination of Barley and Wheat
Source: Plants (Basel). 2021 Dec 11;10(12):2728. doi: 10.3390/plants10122728 (PMC8708003; doi:10.3390/plants10122728)
Supplement: Supplementary file 1 [file plants-10-02728-s001.zip › plants-1483347-supplementary.pdf]

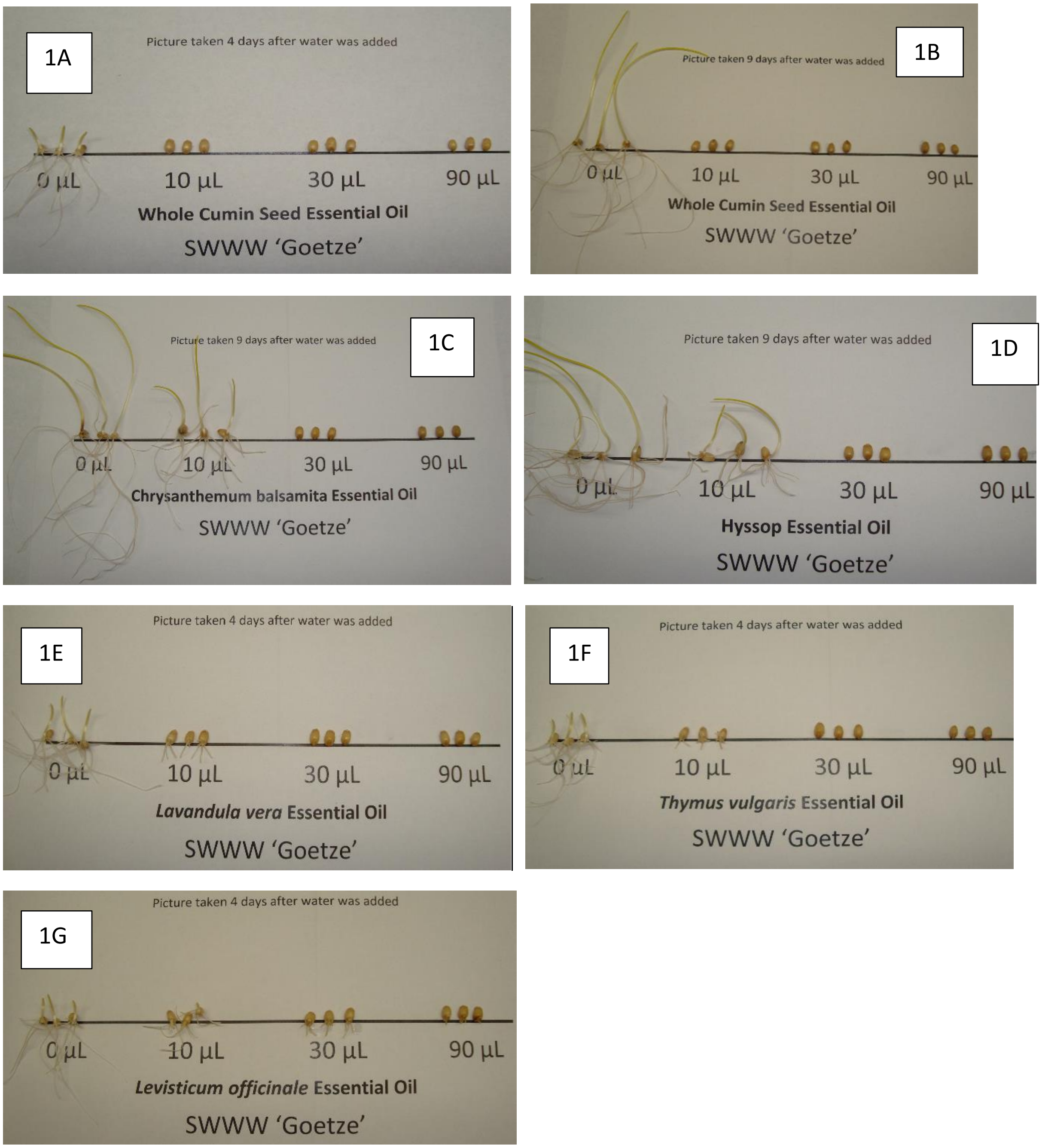

Figure S1. Representative images of germinating wheat in various treatments using the essential oils: 1A and **1B** - *Cuminum cyminum;* **1C**- *Chrysanthemum balsamita;* **1D** - *Hyssopus officinalis;* **1E** - *Lavandula angustifolia;* **1F**- *Thymus vulgaris; and* **1G** - *Levisticum officinale.*

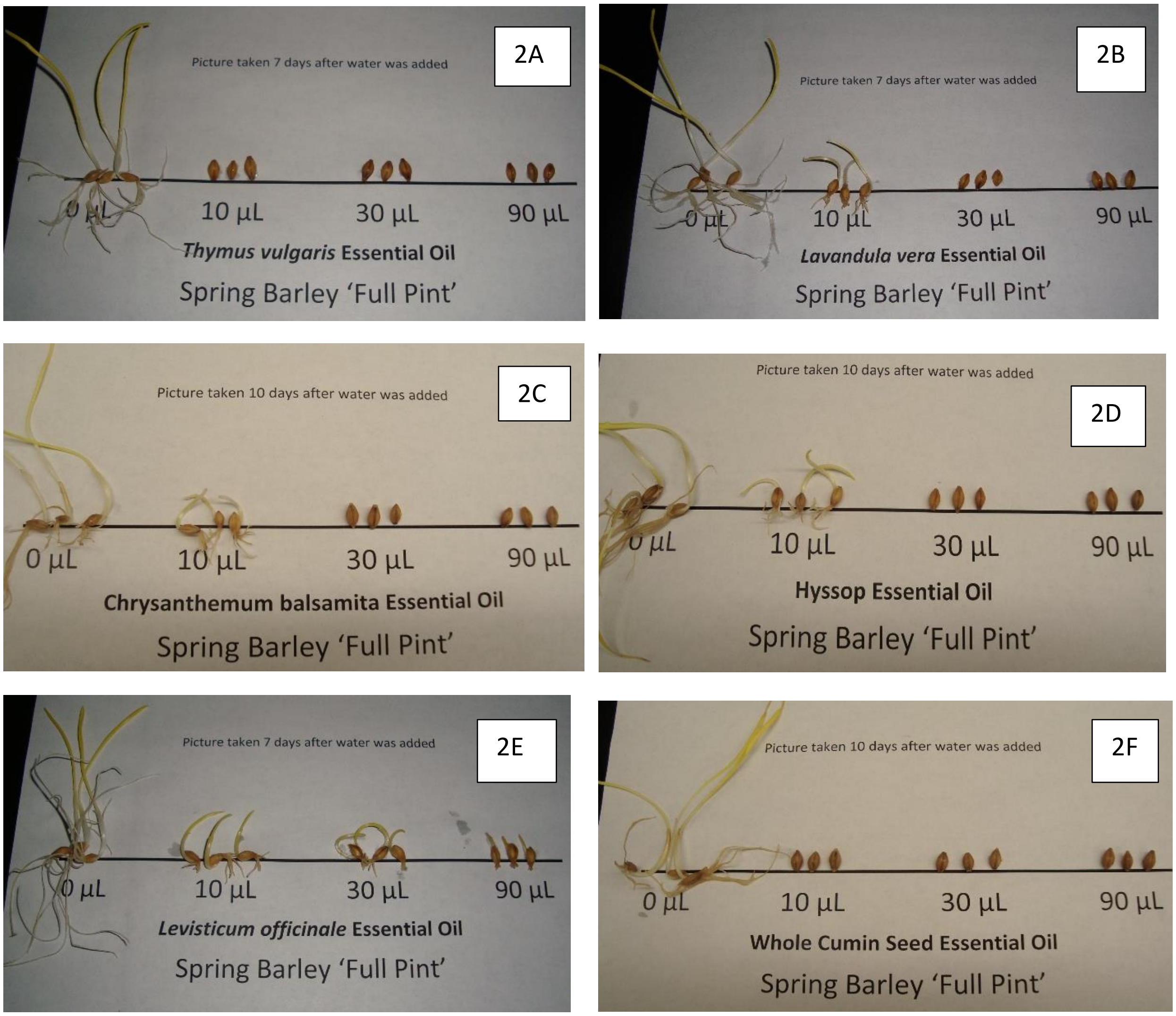

Figure S2. Representative images of germinating barley in various treatments: **2A** *Thymus vulgaris;* **2B** - *Lavandula angustifolia;* **2C** -*Chrysanthemum balsamita;* **2D** - *Hyssopus officinalis;* **2E** - *Levisticum officinale; and* **2F** - *Cuminum cyminum.*
